# Surgeon-General John George Faught

**Published:** 1910-06-25

**Authors:** 


### OBITUARY.

Surgeon-General John George Faught, who died last Sunday at Southsea, in his seventy-eighth year, was an Honorary Surgeon to the King. He was a member of the Royal College of Surgeons, and entered the Medical Department of the Army in 1855 as assistant surgeon, and retired on December 15, 1892, with the rank of surgeon-major-general. During the Ashanti war of 1874 he served as sanitary officer at Cape Coast Castle. For his services in the Afghan and African wars he was decorated with the Indian and Ashanti medals. He afterwards served during the Egyptian war of 1882, and received the medal and Khedive's bronze star. Again in 1884-85 he was principal Medical Officer to Sir Charles Warren in Bechuanaland, and in Zululand in 1888 under Sir W. Smyth.

